# Triage in the mass casualties in pre-hospital emergency 

**Published:** 2019-08

**Authors:** Abbas Dadashzadeh, Jafar Khani, Mostafa Soleymani

**Affiliations:** ^*a*^PhD in Nursing, Tabriz University of Medical Sciences, Tabriz, Iran.; ^*b*^MSc Emergency Nursing Student, Zanjan University of Medical Sciences, Zanjan, Iran.

## Abstract

**Background:**

In the everyday life, a large number of pre-hospital emergency missions are including the injurers caused by traumatic events. Meanwhile, the immediate response to the injurers of the mass casualties is of particular importance due to their large number and also the distance to the medical centers. Using of a proper triage scale has a major role in prioritizing the injuries resulted from mass casualty events in order to treat and transfer them to health centers and also in the management of the scene and improving the quality of care of traumatic injuries. Therefore, the purpose of this study is to describe the various methods of triage in the scene, so that we can present a suitable triage scale for prioritizing injuries in mass casualty for pre-hospital emergency.

**Methods:**

This study is an overview one and is performed by using of databases and library resources and words including triage, pre-hospital emergency, mass casualty, crisis and emergency medical technician.

**Results:**

Based on the surveys, a variety of triage methods in the scene were found including Simple triage and rapid treatment (START), Jump start, triage sieve or Major Incident Medical Management and Support (MIMMS), Pediatric Triage Tape (PTT), Move, Assess, Sort, and Send (MASS), Care Flight, Sort, Assess, Lifesaving interventions, and Treatment and/or Transport (SALT), Revised Trauma Score (RTS), Pediatric Trauma score (PTS).

**Conclusions:**

In the literature, the use of different triage scales has been recommended in the crisis, the scene of the accident and trauma. By considering the context, structure and dispersion of mass casualty in the country, it isn’t known yet which one of these scales is operational and applicable. Therefore, for the patients in mass casualty, it is suggested that an appropriate and applicable triage scale should be designed for pre-hospital emergency operations.

**Keywords:**

Triage in the scene, Pre-hospital emergency, Mass casualty

